# Tumor-targeting bacteria as immune stimulants – the future of cancer immunotherapy?

**DOI:** 10.1080/1040841X.2024.2311653

**Published:** 2024-02-12

**Authors:** Alexandra M. Mowday, Jella M. van de Laak, Zhe Fu, Kimiora L. Henare, Ludwig Dubois, Philippe Lambin, Jan Theys, Adam V. Patterson

**Affiliations:** a Auckland Cancer Society Research Centre, School of Medical Sciences, University of Auckland, Auckland, New Zealand; b Maurice Wilkins Centre for Molecular Biodiscovery, University of Auckland, Auckland, New Zealand; c The M-Lab, Department of Precision Medicine, GROW—Research School of Oncology and Reproduction, Maastricht University, Maastricht, The Netherlands; d Malaghan Institute of Medical Research, Wellington, New Zealand

**Keywords:** Tumor-targeting bacteria, cancer, immunotherapy, immune stimulant, immune response

## Abstract

Cancer immunotherapies have been widely hailed as a breakthrough for cancer treatment in the last decade, epitomized by the unprecedented results observed with checkpoint blockade. Even so, only a minority of patients currently achieve durable remissions. In general, responsive patients appear to have either a high number of tumor neoantigens, a preexisting immune cell infiltrate in the tumor microenvironment, or an ‘immune-active’ transcriptional profile, determined in part by the presence of a type I interferon gene signature. These observations suggest that the therapeutic efficacy of immunotherapy can be enhanced through strategies that release tumor neoantigens and/or produce a pro-inflammatory tumor microenvironment. In principle, exogenous tumor-targeting bacteria offer a unique solution for improving responsiveness to immunotherapy. This review discusses how tumor-selective bacterial infection can modulate the immunological microenvironment of the tumor and the potential for combination with cancer immunotherapy strategies to further increase therapeutic efficacy. In addition, we provide a perspective on the clinical translation of replicating bacterial therapies, with a focus on the challenges that must be resolved to ensure a successful outcome.

## Graphical Abstract

**Figure:**
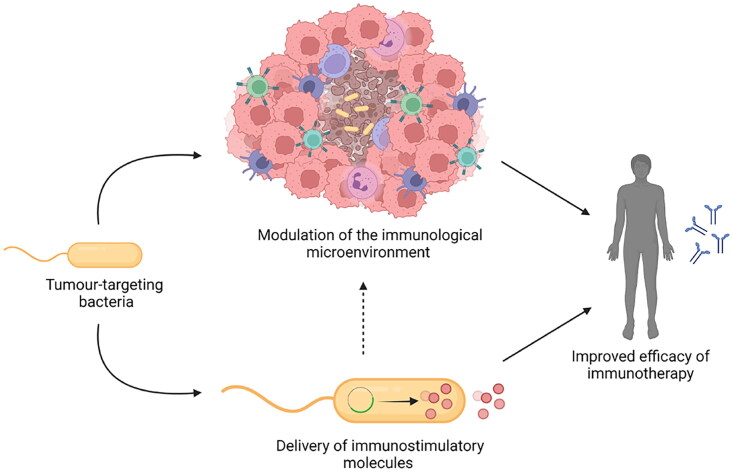

## 1. Bacterial infection as the first cancer ‘immunotherapy’

Observations of a relationship between coincidental infection and spontaneous tumor regression date back to the eighteenth century, if not earlier. Establishment of suppurating sores, application of septic dressings to ulcerated tumors, and deliberate introduction of infections such as gangrene or syphilis to patients with tumors were crude early forms of immunotherapy that became widely known and accepted at the time (Hoption Cann et al. 2003). One of the most prominent scientists to take advantage of these observations was William B. Coley, who inoculated ten patients that had inoperable tumors with *Streptococcus pyrogenes* (Coley 1910). Whilst some tumor regressions were observed, the degree of the induced infection varied wildly between patients and was occasionally fatal (Coley 1910). Due to this unpredictability, he elected to switch to using a solution containing toxins filtered from two heat-killed bacteria, *S. pyrogenes* and *Serratia marcescens* (Coley 1910). Coley’s toxins successfully produced complete and prolonged regression of advanced disease in a variety of tumor types, most notably sarcoma, but the therapy did not work consistently enough to bring benefit to the majority of patients (Kucerova and Cervinkova 2016). He emphasized that induction of fever was crucial to achieve therapeutic benefit, and a retrospective study confirmed that a greater five-year survival rate was reported in patients that exhibited this symptom (Nauts 1959). Although Coley never completely understood the mechanism for how his bacterial extracts functioned, further elucidation and development of the connection between the immune system and cancer would come years later. Coley’s toxins are now believed to have worked, at least in part, by binding to and stimulating toll-like receptors (TLRs) on immune cells (Orange et al. 2016).

Concurrent with the development of Coley’s toxins was the identification of a particular *Mycobacterium bovis* strain, “Bacille de Calmette et Guérin” (BCG), for use in superficial bladder cancer (Meyer et al. 2002). BCG was originally developed as a vaccine for tuberculosis but following the work of Coley and the development of syngeneic animal models, it was demonstrated that mice infected with BCG showed increased resistance to challenge with transplantable tumors (Old et al. 1959; Zbar et al. 1971). The clinical use of BCG as a therapy for bladder cancer began in 1976 (Morales et al. 1976), and in 1990 it became the first and only FDA-approved live bacterial therapeutic vaccine for cancer. The exact mechanism of anti-tumor activity has not been completely determined but is thought to be the result of an interplay between direct effects on tumor cells by BCG infection and the host’s immune response (Kawai et al. 2013; Han et al. 2020). Today, intravesical BCG therapy remains the standard of care for preventing relapse in high-grade noninvasive bladder cancer post-surgery.

Since 2010, cancer therapy has undergone a paradigm shift toward harnessing an anti-tumor immune response as a fundamental treatment strategy. Stimulated by the unprecedented success achieved by checkpoint blockade, the concept of using systemically administered bacteria to treat cancer is undergoing a renaissance. Bacteria offer many advantages over traditional pharmaceutical products due to their diversity, tumor selectivity, non-overlapping side-effect profiles, and ability to be genetically engineered to synthesize and release specific (macro)molecules. Their tendency for ‘tumor agnostic’ properties means that their effectiveness is not directly affected by the genomic landscape of a tumor, and *a priori* knowledge of the identity of the tumor-derived neoantigens unique to a given patient/tumor is not required. This is in direct contrast to other immunotherapy strategies such as CAR T-cell therapy, where heterogeneity between patients requires individualized antigen selection, an often complicated and costly process (Liu et al. 2019). In this review, we will discuss exogenous tumor-targeting bacteria, their modulation of the immunological microenvironment in the tumor following colonization, and the potential for use as immune stimulants in combination with cancer immunotherapy strategies to improve effectiveness. Bacteria of the tumor-associated microbiome (Nejman et al. 2020) and gut microbiome (Gopalakrishnan et al. 2018) have been recently reviewed in the context of cancer therapy elsewhere.

## 2. Mechanisms of tumor targeting used by bacteria

Many bacterial strains have been shown to accumulate and proliferate preferentially within solid tumors using a variety of unique mechanisms (discussed in detail by Morrissey et al. 2010 and Forbes 2010). In general, the initial number of bacteria delivered to normal and tumor tissue is similar, but bacteria in the circulation and/or normal tissues are rapidly cleared whilst bacteria in the tumor are able to proliferate and thus amplify to numbers greatly exceeding the initial input dose (Zhou et al. 2018). This selective proliferation is generally extracellular, and thought to be enabled by exploiting the unique characteristics of the tumor microenvironment, including immunosuppression, hypoxia, necrosis, and metabolite enrichment or deficiency. For example, *Clostridium* species are spore-forming obligate anaerobes, and germination from inert spores into saprophytic bacteria is restricted to regions of tumor necrosis where sufficient anoxia is present (Fabricius et al. 1993; Mowday et al. 2016). In theory, the tumor-selective germination of this species should allow for intravenous administration with minimal systemic toxicity. *E. coli* and *Salmonella* species are facultative anaerobes, so have some selectivity for accumulation in the hypoxic and necrotic regions of solid tumors but can also colonize normal tissues to an extent (Wei et al. 2008). In recent years, the tumor specificity of first-generation *Salmonella* strains has been significantly increased using genetic modification (Liang et al. 2019). A potential advantage to the use of facultative anaerobes is their ability to colonize smaller metastatic deposits. In addition, *Salmonella* species have demonstrated preferential chemotaxis toward cancer cells (Kasinskas and Forbes 2006). Interestingly, *Listeria* species have a unique way of targeting the tumor through involvement of the host immune system, providing them with the ability to grow intracellularly. *Listeria* can internalize *via* phagocytosis into antigen presenting cells (APCs) and myeloid-derived suppressor cells (MDSCs), which then deliver the bacteria to the immunosuppressive tumor microenvironment. Here, they are protected from immune clearance and can go on to infect tumor cells (Paterson and Maciag 2005; Chandra et al. 2013). Genetic modification has also been used to improve the tumor tropism of bacteria. For example, essential genes have been placed under the control of promoter elements responsive to hypoxia (Yu et al. 2012) or low pH (Flentie et al. 2012), and bacteria have been engineered to express peptides on the outer membrane that selectively bind to integrins that are over-expressed on cancer cells (Park et al. 2016).

## 3. Modulation of the immunological microenvironment in the tumor

Cancer immunotherapy and the emergence of immune checkpoint blockade has been widely hailed as a breakthrough for cancer treatment in the last decade, changing the paradigm for cancer therapy (Couzin-Frankel 2013). It functions to disrupt cancer cell evasion of the immune response to produce heightened and sustained anti-tumor immunity, generating unprecedented results in a variety of cancer types, including melanoma (Robert et al. 2019), lung (Herbst et al. 2016), and head and neck (Mehra et al. 2018). Unfortunately, only a minority of patients will achieve long-term durable remissions (Robert 2020). The effectiveness of cancer immunotherapy strategies can depend on the presence of a pre-existing immune response (Ochoa de Olza et al. 2020). However, many tumor microenvironments are immunosuppressive, defined as either ‘cold’ (absence of T-cells in both the tumor center and invasive margins), ‘altered-suppressed’ (low density of T-cell infiltration into the tumor but an immunosuppressive environment limits further recruitment or expansion) or ‘altered-excluded’ (T-cell density at the tumor margins but no infiltration into the center) (Camus et al. 2009). Multiple mechanisms can drive this observed immunosuppression, including high levels of immunosuppressive cytokines that impair immune cell functions (e.g. IL-10, TGF-β, Li et al. 2020), recruitment and expansion of immunosuppressive cell types (e.g. Tregs Togashi et al. 2019), tumor hypoxia (Fu et al. 2021), transcriptional alterations (Routh et al. 2020), and sustained expression of inhibitory receptors that lead to an exhausted, unresponsive T-cell phenotype (e.g. PD-1, CTLA4, TIM3 Zhang et al., 2020).

**Figure 1.**
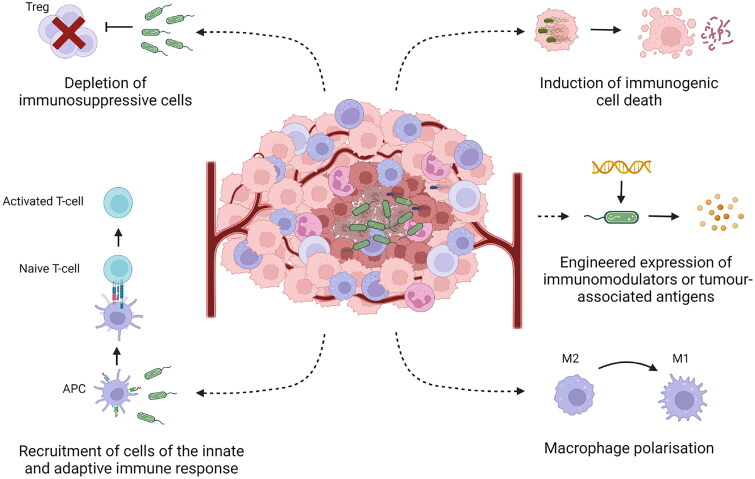
A Summary of the mechanisms by which tumor-targeting bacteria can modulate the immunosuppressive tumor microenvironment (see text for details).

Selective colonization of the tumor by exogenous bacteria has the potential to serve as an approach to overcome an immunosuppressive tumor microenvironment, improving the effectiveness of immunotherapy for more people. For example, bacterial colonization could induce immunological cell death, stimulate innate immune responses (adjuvanticity), and recruit cytotoxic immune cells to the tumor microenvironment. In addition, genetically modified strains could also deliver molecules to the tumor that are able to modulate the immune response or induce immunogenicity through expression of tumor-associated antigens (TAAs). These concepts are discussed in more detail below and are summarized in Figure 1.

### 3.1. Tumor-selective bacterial infection recruits immune cells to the tumor microenvironment

The innate immune system is the first line of defence against invading pathogens. It relies on detection of evolutionarily conserved motifs associated with pathogen infection (pathogen associated molecular patterns, PAMPs) by a large family of pattern recognition receptors (PRRs) that signal to the host the presence of infection (Akira et al. 2006). Examples of bacterial PAMPs include components of the cell wall such as peptidoglycans (Yoshimura et al. 1999) or lipopolysaccharides (LPS) (Mogensen 2009), flagellum (Hayashi et al. 2001), and bacterial DNA (CpG-DNA) (Ahmad-Nejad et al. 2002). They are detected by one of the five major sub-families of PRRs; the Toll-like receptors (TLRs) (Kawai and Akira 2010), the nucleotide-binding oligomerization domain (NOD)- Leucin Rich Repeats (LRR)-containing receptors (NLRs) (Kanneganti et al. 2007), the C-type lectin receptors (CLRs) (Geijtenbeek and Gringhuis 2009), the retinoic acid-inducible gene I (RIG-I)-like receptors (RLRs) (Yoneyama and Fujita 2007), and the absent in melanoma 2 (AIM2)-like receptors (ALRs) (Fernandes-Alnemri et al. 2009). PRR-induced intracellular signaling cascades result in the induction of a multitude of different pathways, which together orchestrate the early host response to infection. The major pathways activated include nuclear factor-κB (NF-κB), mitogen-activated protein kinase (MAPK), the type I interferon (IFN) response, and inflammasome assembly (Li and Wu 2021). Downstream effects produce pro-inflammatory and anti-microbial responses designed to eliminate or contain pathogens, including the synthesis of cytokines and chemokines, in addition to the induction of infected cell death in an attempt to halt pathogen spread (Bortoluci and Medzhitov 2010).

The cellular elements of this acute inflammatory response that migrate from the peripheral blood to the site of infection include monocytes, neutrophils, basophils, eosinophils and NK cells (Iwasaki and Medzhitov 2004). Infiltrating monocytes can differentiate into either tissue-resident macrophages or dendritic cells (DCs) (Yang et al. 2014). DCs are myeloid cells that can capture microbial antigens, process them, present them to naïve T-cells as peptides bound to major histocompatibility complex molecules (MHC) on their cell surface, and express co-stimulatory molecules upon maturation (Banchereau and Steinman 1998). This peptide-MHC complex is recognized by the T-cell receptor and their interaction leads to the activation and differentiation of naïve T-cells into effector T-cells. These include cytotoxic T-cells (CTLs, CD8^+^), which may directly induce the death of microbe-infected host cells, and helper T-cells (CD4^+^), which can enhance DC function and subsequently the CD8^+^ T-cell response *via* CD40-CD40L interaction. Effector T-cells can also form memory T-cells to help to provide immunological memory, enabling them to respond more rapidly and effectively against foreign pathogens when the same antigen is encountered again (Seder and Ahmed 2003). The function of DCs can also be enhanced *via* direct stimulation of the PRRs on the cell surface, leading to upregulation of co-stimulatory molecules and production of cytokines such as type I interferons, which in turn may enhance the CD8^+^ T-cell response against tumor-specific neoantigens. DCs therefore serve as a fundamental link between the innate and adaptive immunity (Banchereau and Steinman 1998; Petersen et al. 2010). Macrophages and granulocytes (i.e. neutrophils, basophils, and eosinophils) are other types of myeloid cells that are also crucial in innate immunity which can recognize, engulf, and destroy pathogens. Like DCs, macrophages are also APCs that possess the ability to present antigens to T-cells in the context of MHC molecules, provide the necessary co-stimulatory signals, and produce pro-inflammatory cytokines required for effective T-cell activation (Guerriero 2019).

Recruitment of immune cells to the tumor microenvironment following selective colonization by bacteria has been observed with a variety of species. For example, *Clostridium* species have produced innate immune cell infiltration (mainly neutrophils and NK cells) in the tumor following treatment in a variety of models, including subcutaneous mouse and rabbit allografts (Agrawal et al. 2004; Maletzki et al. 2010), orthotopic brain tumors (Staedtke et al. 2015) and naturally occurring canine tumors (Krick et al. 2012; DeClue et al. 2018). Often, these inflammatory cells accumulated at the border between the proliferative and necrotic areas of the tumor when colonized by the most clinically advanced strain, *C. novyi*-NT (Agrawal et al. 2004). Interestingly, the local accumulation of neutrophils has been observed to impede bacterial spread and prevent complete oncolysis (Staedtke et al. 2022). Re-challenge of mice that were cured of their tumors by *Clostridium novyi*-NT infection were resistant to regrowth of repeat tumor inoculation in 8/10 animals, with this effect shown to be mediated by CD8^+^ T-cells (Agrawal et al. 2004) indicating establishment of durable immune memory. *E. coli* species have also demonstrated CD8^+^-mediated anti-tumor immune responses in this manner (Stern et al. 2015).

Like *Clostridium* and *E. coli*, *Salmonella* colonization can lead to increased recruitment of immune cells to the tumor, including neutrophils, macrophages, NK cells, DCs, B-cells, CD8^+^ T-cells, and CD4^+^ T-cells (Vendrell et al. 2011; Murakami et al. 2018; Kim et al. 2015; Grille et al. 2014; Hernández-Luna and Luria-Pérez 2018). Interestingly, *Salmonella* can enhance antigen presentation by DCs through upregulation of connexin 43 on tumor cells to form new gap junctions, allowing transfer of pre-processed antigenic peptides from the tumor cells directly to DCs (Saccheri et al. 2010). This indicates that activated CD8^+^ T-cells could play an important role in the inhibition of tumor growth that is observed during tumor-targeted therapy by *Salmonella* (Hong et al. 2013). Tumor-targeted infection by recombinant *Listeria* can also mount a protective tumor-specific CD8^+^ T-cell response (Deng et al. 2018). Overall, these data indicate that tumor-selective colonization by bacteria can promote the infiltration of multiple types of immune cells into the tumor microenvironment, with the potential to enhance the innate and adaptive anti-tumor immune responses that contribute to tumor regression.

### 3.2. Tumor-targeting bacteria can be oncolytic and induce immunogenic cell death

Immunogenic cell death (ICD) is defined as a form of cell death that elicits an immune response (Kroemer et al. 2013). ICD is accompanied by the exposure, active secretion or passive release of various damage-associated molecular patterns (DAMPs) that bind to the PRRs expressed on APCs to induce activation of both innate and adaptive immune responses (Kepp et al. 2014). However, DAMPs cannot initiate this response unless the dying cell displays an increased antigenicity, e.g. increased expression of host genes that have mutated during tumourigenesis (tumor neoantigens) (Galluzzi et al. 2017). Thus, ICD relies on both the antigenicity of the cell and adjuvanticity (conferred by DAMPs) (Galluzzi et al. 2017). There are three major DAMPs that are associated with ICD: cell surface-exposed calreticulin, extracellular ATP, and high mobility group box 1 protein (HMGB1) (Kepp et al. 2014). Accumulating evidence suggests that monitoring DAMPs or DAMP-associated stress responses in cancer patients may have prognostic value (Fucikova et al. 2015), positively correlating with favorable disease outcome in a number of cancer types including colorectal (Peng et al. 2010), esophageal (Suzuki et al. 2012), breast (Arnold et al. 2013) and ovarian cancer (Kasikova et al. 2019). Induction of ICD provides a new opportunity to improve the effectiveness of cancer treatment, causing a tumor-specific immune response that can result in long-term clinical benefits. For example, ICD is thought to be responsible for the radiation-induced abscopal effect, whereby regression of non-irradiated metastatic lesions occurs at a site distant from the primary site of irradiation (Rodríguez-Ruiz et al. 2018). Some classical chemotherapies are known to induce ICD, including doxorubicin (Casares et al. 2005), oxaliplatin (Tesniere et al. 2010) and cyclophosphamide (Schiavoni et al. 2011), whilst newer methods for inducing ICD have also been developed such as oncolytic virotherapy (van Vloten et al. 2018) and near infrared photoimmunotherapy (Nakajima et al. 2018). Overall, this suggests that induction of the ICD holds significant promise for cancer therapy.

Colonization of tumor tissues by bacteria can be oncolytic, with the potential to produce ICD and provoke a potent and sustained anti-tumor immune response. Attenuated strains of *Salmonella* that have been engineered to target tumors are directly oncolytic, proliferating inside infected tumor cells until they burst and die (Uchugonova et al. 2015). Induction of ICD in tumors following *Salmonella* treatment has been demonstrated in the syngeneic 4T1 model, with a significant increase in the DAMP calreticulin (per mg of tumor tissue) compared to untreated tumor-bearing controls, which was associated with reduction in tumor growth and prolonged survival (Chirullo et al. 2015). Similarly, the presence of extracellular ATP has been observed following *in vitro* infection of MC38 cells with *Salmonella* (Phan et al. 2015). In contrast, *Clostridium* species secrete proteolytic enzymes, hemolysins, and lipases that can damage tumor cell membranes and cause cell lysis in an indirect manner (Bettegowda et al. 2006; Cheong et al. 2006). It is conceivable that this process may evoke ICD, although this has not been definitively determined. *Listeria* species can kill tumor cells directly, through activation of nicotinamide adenine dinucleotide phosphate oxidase (NADPH oxidase) and increased levels of intracellular calcium (Kim et al. 2009) resulting in the production of high levels of reactive oxygen species (ROS) and oxidative stress, the presence of which can increase DAMP emission and ICD (Krysko et al. 2012). In fact, most organisms produce ROS in some way when responding to pathogen infection in an attempt to prevent colonization of tissues (Spooner and Yilmaz 2011), suggesting that this means of ICD production could be applicable to other tumor-selective bacteria. In summary, the inherent oncolytic properties of bacteria give them the capacity to produce ICD in the tumor, providing robust adjuvanticity to dying cells and favoring recruitment of APCs to generate an anti-tumor immune response.

### 3.3. The presence of bacteria removes immunosuppressive cell types from the tumor microenvironment

An immunosuppressive tumor microenvironment is one of the major factors promoting tumor progression, by establishing favorable conditions that facilitate growth and metastasis (Whiteside 2008). Such an environment also presents a challenge for the efficacy of cancer immunotherapy (Hegde et al. 2016). An immunosuppressive tumor milieu is, in part, characterized by the recruitment of key immunosuppressive cell subsets, including myeloid-derived suppressor cells (MDSCs), regulatory T-cells (Tregs), and tumor-associated macrophages (TAMs, discussed in further detail in section 3.4 below) (van der Woude et al. 2017). These cells function as effectors to supress the immune response *via* production of numerous immunosuppressive cytokines such as vascular endothelial growth factor (VEGF) (Lee et al. 2020), TGF-β (Syed 2016), IL-10 (Zhao et al. 2015), and enzymes such as indoleamine 2,3-dioxygenase (IDO) (Zhai et al. 2020). In addition to dampening responses against microbes, allergens, and tumors, Tregs are essential at preventing autoimmunity to maintain the homeostasis of the immune response (Vignali et al. 2008). However, tumor-associated Tregs can directly promote tumor immune evasion through a number of humoral and cell-cell contact mechanisms. These include production of suppressive cytokines (e.g. IL-10 and TGF-β), consumption of IL-2 (crucial for promoting differentiation of effector and memory T-cells), and high expression of the inhibitory molecules such as cytotoxic T lymphocyte-associated protein 4 (CTLA-4) (Tanaka and Sakaguchi 2017). MDSCs are a heterogeneous population of immature myeloid cells, capable of changing their functional status in response to a variety of environmental signals such as cytokines or growth factors (Gabrilovich and Nagaraj 2009). The immunosuppressive activity of MDSCs in tumors mainly includes inducing the differentiation and expansion of Tregs, depleting or sequestering amino acids critical for T-cell functions, inducing oxidative stress, and blocking of lymphocyte homing (Groth et al. 2019).

There is accumulating evidence that depletion of Tregs and/or differentiation of MDSCs into mature myeloid cells can diminish the immunosuppressive properties of the tumor microenvironment and consequently enhance the anti-tumor immune response. For example, the use of an anti-CD25 antibody to differentially target and deplete Tregs in the tumor resulted in demonstrable regressions in xenograft models (Onizuka et al. 1999) and all-trans-retinoic acid (ATRA) has been shown to promote differentiation of MDSCs into DCs to improve antigen-specific T-cell responses (Mirza et al. 2006). Bacterial colonization of the tumor microenvironment has been used to try and overcome the immunosuppressive function of Tregs and MDSCs in a similar way. Intratumoural *Salmonella* injections have caused demonstrable reductions in Tregs in the tumor or tumor draining lymph nodes, which was associated with an inhibitory effect on tumor growth (Vendrell et al. 2011; Hong et al. 2013). Additionally, expansion of a population of myeloid cells secreting TNF-α was observed, which contributes to the anti-tumor effect during *Salmonella* infection (Hong et al. 2013). The effect of *Listeria* on the immunosuppressive cells of the tumor microenvironment has also been reported. A reduction in frequency and near complete loss of suppressive activity in MDSC and Tregs was observed following treatment of the tumor with a strain of *Listeria (*Wallecha et al. 2013). This effect was found to be associated with reduced expression of Arginase-1 (a key marker of immunosuppressive macrophages) in MDSCs and IL-10 in Tregs. These data demonstrate the potential for bacteria to attenuate the frequency of the immunosuppressive cell populations selectively in the tumor microenvironment, eliciting anti-tumor immune responses and improving clinical prognosis without inducing deleterious autoimmunity.

### 3.4. Bacterial colonization of the tumor modifies macrophage polarization

Tumor-associated macrophages (TAMs) are derived from circulating monocytes that are recruited to the tumor microenvironment by tumor-derived chemokines such as CCL2 (Sica et al. 2006). TAMs demonstrate phenotypic plasticity and can have a dual supportive and inhibitory influence on tumors, depending on a variety of factors including stage, tissue type, and the host microbiota (Bercovici et al. 2019). In nascent tumors, there is evidence that so-called M1-like or ‘classically activated’ macrophages in the tumor can directly mediate extracellular killing of tumor cells, contributing to the early ‘elimination’ phase of cancer immune surveillance (Pan et al. 2020). The IL-12 secreted by these M1-like macrophages also plays an important role in Th1 polarization, whereby CD4^+^ T-cells become restricted to producing a distinct set of pro-inflammatory cytokines, including IFN-ɣ and IL-2 (Muraille et al. 2014). Subsequently, tumor progression is associated with a skewing of TAM function to elicit an immunosuppressive phenotype (so called M2-like or ‘alternatively activated’ macrophages), which seem to be a common feature of most tumors (Mantovani et al. 2017). These M2-like macrophages can influence the intrinsic properties of tumor cells and cells of the tumor microenvironment to promote tumor progression. Examples of which include the expression of growth factors to stimulate tumor cell proliferation and angiogenesis, production of proteolytic enzymes that digest the extracellular matrix to promote dissemination and metastasis and providing a supportive niche for metastatic tumor cells at distant sites (Mantovani et al. 2017).

Bacterial infection can modify the balance of M1/M2 polarization in the tumor, re-educating macrophages to a more classical M1-like phenotype, with increased expression levels of MHC-II molecules required for effective antigen presentation. This has been demonstrated in TAMs that have phagocytosed *Listeria* in an ID8 ovarian cancer model (Lizotte et al. 2014). Following infection, they were reprogrammed to express co-stimulatory molecules such as CD80 and CD86, which act to amplify the initial activating signals provided to T-cells, thus reversing the ability of this TAM population to suppress T-cell function. The infected TAMs could also lyse tumor cells directly through inducible nitric oxide synthase (iNOS) mediated production of nitrous oxide. A similar phenomenon has been observed in tumors colonized with *Salmonella* (Pangilinan et al. 2021). Increased secretion of HMGB1 in tumors following *Salmonella* infection in two melanoma models was determined to be responsible for polarization of macrophages to an M1-like phenotype, with a corresponding increase in M1-like markers iNOS and interleukin 1 beta (IL-1β) observed. Gene expression analysis in another study corroborated the increase in iNOS in tumor myeloid cells following *Salmonella* infection, in addition to a decrease in Arg-1 (Kaimala et al. 2014). Overall, this suggests that selective bacterial colonization of the tumor microenvironment can increase the proportion of M1-like macrophages, promoting their anti-tumor functions of direct tumor cell lysis and enhanced tumor antigen presentation through the increased expression of MHC-II molecules.

## 4. Delivery of immunomodulators to the tumor microenvironment

**Figure 2.**
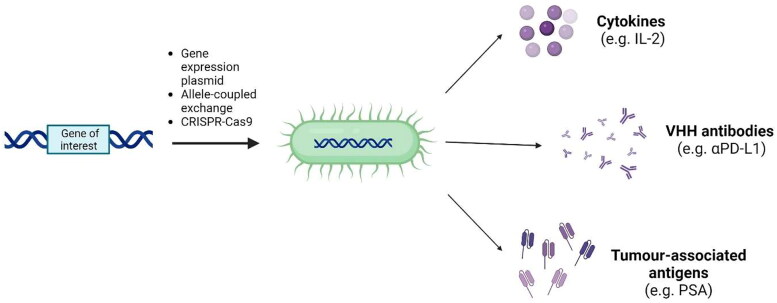
Tumor-targeting bacteria can be genetically modified to express immunomodulatory molecules selectively in the tumor microenvironment, further increasing their therapeutic potential.

Tumor-targeting bacteria can also be genetically engineered to deliver immunomodulatory molecules selectively to the tumor, further increasing their therapeutic potential (Figure 2). Recent developments in recombinant DNA technology have accelerated the development of these strains, but the potential for successful genetic modification still varies between species and can often depend on the availability of compatible and well-characterized genetic tools, the ease of genetic alteration, and the tolerance of the host for any modified genes and/or protein structures. In the past, recombinant bacterial strains would often express the gene of interest from an autonomous plasmid, which usually contained an antibiotic resistance marker (Liu et al. 2008; Xu et al. 1998; Gurbatri et al. 2020). These strains usually demonstrated segregational instability, had the risk for horizontal gene transfer (Popov et al. 2009; Thomas and Nielsen 2005), and were not suitable for clinical development. Precise integration of heterologous expression constructs into the bacterial chromosome without the use of antibiotic resistance markers is now preferred for safety and the stability of gene expression and is achievable through various techniques including Allele-coupled exchange (Heap et al. 2014) and the use of CRISPR-Cas9 technology (Kubiak et al. 2021; Zhang et al. 2023).

Localized delivery of immunomodulatory molecules to the tumor microenvironment by tumor-targeted bacterial strains has the potential to minimize systemic toxicity, thus improving the therapeutic index of these agents. In principle, it may be possible to generate active concentrations in the tumor at levels which are unachievable by systemic injection. Various cytokines have been successfully expressed from bacteria and secreted as biologically active agents, including IFN-ɣ (Yoon et al. 2017), IL-18 (Loeffler et al. 2009), IL-2 (Kubiak et al. 2021; Barbé et al. 2005; Gniadek et al. 2020), and GM-CSF (Gurbatri et al. 2020). Of these, an IL-2 expressing strain of *Salmonella* has progressed the furthest. Promising pre-clinical efficacy in a canine model of osteosarcoma (Fritz et al. 2016) led to a Phase I single-dose clinical trial in human patients with metastatic gastrointestinal tumors (Gniadek et al. 2020). Recently reported results indicated that whilst no toxicities or adverse events were observed, there was no survival advantage. However, a significant increase in circulating NK and NKT cells following treatment suggested that a multi-dose regimen could be used to provide therapeutic benefit.

Functional single-chain antibodies (VHH antibodies) have been expressed from tumor-targeting bacteria with some success, allowing for selective production of more complex molecules in the tumor microenvironment. *E. coli* Nissle 1917 has been engineered for controlled production and intratumoural release of VHH antibodies against PD-L1 and CTLA-4 (Gurbatri et al. 2020). An equal stochiometric mixture of bacteria expressing antibodies against each protein produced an increase in activated CD8^+^ and CD4^+^ T-cells in the tumor, with significant therapeutic efficacy and an increase in survival benefit compared to control strains in an A20 B-cell lymphoma tumor model. This strain of *E. coli* has also been utilized as a platform to produce the STimulator of INterferon Genes (STING)-agonist cyclic di-AMP (CDA), delivery of which induced a variety of pro-inflammatory cytokines and resulted in tumor rejections for 30–80% of animals bearing B16.F10 or A20 tumors (Leventhal et al. 2020). *Clostridium* strains have also been engineered to produce VHH antibodies specific for human HIF-1α (Groot et al. 2007), suggesting that production of antibodies against more immunologically relevant targets such as PD-L1, CTLA-4, CDA or TIM-3 could also be possible in the future.

In an attempt to stimulate an adaptive immune response, bacteria have often been engineered to secrete tumor-associated antigens (TAAs) or present TAA on their surface. For example, *Listeria* species are located within the cytoplasm of infected cells and therefore have the potential to be explored as a vaccine vector for TAAs. In this situation, TAAs are usually expressed as a fusion protein with a listerial virulence factor such as LLO (Gunn et al. 2001). Use of a fusion protein is thought to increase antigenicity through the presence of a protein degrading peptide signal (PEST sequence) at the amino terminus of LLO (Gunn et al. 2001). Preclinical studies have used *Listeria* to successfully deliver tumor antigens such as HPV-16 E7 (Sewell et al. 2004), PSA (Shahabi et al. 2008), HER-2/neu (Shahabi et al. 2011), and Mage-b (Kim et al. 2008) to cells. In contrast, *Salmonella* species become trapped within the phagosomes of infected cells, but their type III secretion system can be adapted to directly ‘inject’ antigens into the cytosol (Nishikawa et al. 2006). This approach has been used to deliver a cancer-testis antigen (NY-ESO-1) to tumors, eliciting efficient antigen presentation *via* the MHC class I pathway and significant tumor regressions. The concept of ‘molecular mimicry’, where microbial antigens share cross-reactivity with tumor antigens, has been used to stimulate an adaptive immune response through intestinal bacteria (Fluckiger et al. 2020). In this study, oral administration of *E. coli* expressing the TMP1 epitope (encoded by the genome of a *Siphoviridae* bacteriophage) regressed subcutaneous MCA205 tumors, likely due to the homology of TMP1 to the overexpressed PSMB4 tumor antigen. In future, tumor-targeting bacterial strains could be engineered to express antigens such as TMP1 to stimulate anti-tumor immunity-inducing responses from within the tumor microenvironment.

## 5. Combining targeted bacterial infection of the tumor with cancer immunotherapies

Selective colonization of tumor tissues by bacteria has the potential to sensitize tumors to subsequent treatment with immunotherapy and improve rates of response. There are multiple ongoing and early-stage clinical trials that aim to successfully combine tumor-targeting bacteria with immunotherapy, examples of which are indicated in Table 1.

**Table 1.** Examples of tumor targeting bacteria/immunotherapy strategies currently active and/or recruiting for clinical trial (clinicaltrials.gov; accessed 8 January 2024).

Bacterial species	Clinical trial Identifier	Treatment	Cancer type	Phase	Number of enrolled patients	Treatment outcome/s
*Clostridium novyi-NT*	NCT03435952	*Clostridium novyi-NT* (IT) combination with Pembrolizumab (IV)	Solid tumors	I	16 ^A^	Objective response rate = 25% (*n* = 3 PR, *n* = 1 CR) (Nelson et al. 2023)
*Salmonella enterica*	NCT04589234	*Salmonella typhimurium* expressing IL-2 (oral)	Pancreatic	II	60 ^E^	*N* = 1 patient with CR for pulmonary metastases & PR for primary cancer (Saltzman 2021)
	NCT03750071	VXM01 (oral) combination with Avelumab (IV)	Glioblastoma	I/II	28 ^A^	Objective response rate for non-resectable tumors (*n* = 25) = 12% (*n* = 3 PR) (Wick et al. 2022)
	NCT03762291	TXSVN vaccine (oral) against TAA Survivin	Multiple myeloma	I	1 ^A^	N/A
*E. coli Nissle 1917*	NCT04167137	*E. coli* producing CDA (IT) - monotherapy or combination with Atezolizumab (IV)	Solid tumors	I	32 ^A^	SD observed in four patients (Luke et al. 2023)
*Listeria monocytogenes*	NCT03847519	ADXS-503 (IV) combination with Pembrolizumab (IV)	NSCLC	I/II	17 ^A^	Objective response rate = 12% (*n* = 2 PR), *n* = 6 patients with SD (Gerstner et al. 2022)
	NCT03006302	CRS-207 (IV) combination with Pembrolizumab (IV)	Pancreatic	II	40 ^A^	N/A
	NCT05014776	CRS-207 (IV) combination with Pembrolizumab (IV) & Ipilimumab (IV)	Pancreatic	II	20 ^E^	N/A

IV: intravenous; IT: intratumoural; CDA: synthetic cyclic diadenyl monophosphate, a STING agonist; E: estimate; A: actual; PR: partial response; CR: complete response; SD: stable disease; N/A: not available.

The first-in-human study of intratumoural (IT) injection of *C. novyi*-NT in combination with intravenous administration of the immune checkpoint blockade pembrolizumab has produced three partial responses and one complete response in a cohort of sixteen patients enrolled in the study, with no observable dose limiting toxicities. Recruitment of patients is continuing (NCT03435952) (Janku et al. 2020;  Nelson et al. 2023). A trial initiated in October 2020 aims to evaluate an IL-2 expressing strain of *Salmonella* in metastatic pancreatic cancer in combination with the current standard of care (FOLFIRINOX or gemcitabine, NCT04589234). An initial case study in a single patient provided evidence of a reduction in size of the primary tumor and hepatic metastases, with a significant increase in NK cells (Batist et al. 2020). An attenuated strain of *Salmonella* engineered to express the TAA Survivin is also in clinical trial (NCT03762291), as well as attenuated strains in combination with immunotherapy (NCT03750071). Synlogic’s strain of *E. coli* Nissle 1917 engineered to express the STING agonist cyclic diadenyl monophosphate has produced evidence of STING pathway target engagement when used as a monotherapy in a first-in-human-study (NCT041671137) (Luke et al. 2023; Janku et al. 2021). This data supported initiation of the second arm of the trial, in which this bacterium will be combined with the immune checkpoint blockade atezolizumab. Previous clinical trials combining an attenuated form of *Listeria monocytogenes* (CRS-207) with a pancreatic cancer vaccine (GVAX) and chemotherapy (cyclophosphamide) did not show an improvement in survival over single-agent chemotherapy (Le et al. 2019), but this triple combination therapy is now being trialed with the addition of immune checkpoint blockade to the treatment schedule (NCT03006302). Preliminary results on this trial have not yet been reported. A study evaluating an alternative form of attenuated *Listeria monocytogenes* (ADXS-503) in combination with Pembrolizumab is ongoing (NCT03847519).

## 6. Clinical translation

Tumor-targeting bacteria have shown promising results in a variety of preclinical experimental models, but this success has yet to be fully realized in human clinical studies. Their tumor selectivity makes them an ideal vehicle for the delivery of immunomodulatory payloads to the tumor microenvironment, but challenges still exist in developing bacterial therapeutics for widespread clinical use. An obvious concern is patient safety. Replicating bacterial vectors can pose issues around uncontrolled growth and spread beyond the tumor site, and the strains used are often innately pathogenic. In addition, the cancer patients recruited to initial trials with this therapy are typically heavily pretreated and thus are often immunocompromised. Virulence attenuation of pathogenic strains and the ability to use antibiotics to eliminate the burden of infection go some way toward ameliorating these concerns. The ability to noninvasively observe tumor colonization and/or bacterial replication would be of huge translational advantage as a companion diagnostic. For example, genomic insertion of an imaging capable reporter gene could allow for real-time quantitative monitoring of transgene expression as a surrogate for the spatial and temporal distribution of bacterial spread (Mowday et al. 2020; Min et al. 2021; Azizian et al. 2021). Stringent patient selection and monitoring will be required to mitigate undue risk and prevent undesirable outcomes. It is possible that some patients may have preexisting immunity to common bacterial strains such as *Salmonella*, potentially reducing the ability for repeated administration. In this situation, the use of a spore-forming bacterium such as *Clostridium* could be an advantage. The inert endospores of *Clostridium* do not elicit an immune response (Fabricius et al. 1993), therefore multiple consecutive treatment cycles are possible (Theys et al. 2006). *Clostridium’s* preference for necrotic tissue might also prove uniquely advantageous in clinical trials recruiting patients with advanced stage disease, particularly given the correlation between increasing necrotic burden with poor overall survival (Richards et al. 2011).

Another challenge for the translation of bacterial therapeutics toward human studies are the regulatory and commercial issues associated with clinical development. For example, unlike conventional cancer therapeutics, tumor-targeting bacteria can replicate robustly within the tumor microenvironment. Therefore, the administered dose is not always the same as the therapeutic or effective dose. This dose-response relationship is not readily predicted, as it is highly dependent on the composition of the tumor and how well the tumor tissue supports bacterial proliferation. The US Food and Drug Administration (FDA) have recently recognized this issue and drafted new guidance focusing on the commercialization of live biotherapeutic organisms and early clinical trial design issues that are unique to the study of these types of therapies (docket number FDA-2015-D-3399). In addition, noninvasive imaging of the hypoxic and/or necrotic fraction of the tumor using PET or MRI approaches may help to select patients who would benefit most (Gagel et al. 2004; Egeland et al. 2011).

Good Manufacturing Practise (GMP) procedures for replicating therapies can also present major hurdles to clinical translation. A detailed description of all products and procedures used during the derivation, production, and purification of the product needs to be provided, including information on the seed stock, physical properties, growth characteristics, and genetic makeup. Post-manufacturing, tests to determine the safety, identity, purity, and potency of the final product are essential. Again, docket number FDA-2015-D-3399 provides some guidance on these aspects. Additionally, it might be possible to use the insights gained from the manufacture of probiotics to improve production processes and thus clinical translation of tumor-targeting bacteria. For example, *Clostridium butyricum* MIYAIRI588® spores (CBM 588®) are produced using submerged anaerobic fermentation (Miyarisan Pharmaceutical Co. Ltd., n.d). This process is already utilized commercially to create pickles, sauerkraut and vinegar, negating the need for *de novo* development of a production strategy. In addition, use of a liquid broth allows for the easy generation of spores on a very large scale (in comparison to agar plates, for example), with the potential to minimize batch-to-batch differences.

“Shedding” or excretion/release of bacterial products from the patient’s body into the environment poses additional regulatory concern. The US FDA provides guidance on how and when shedding data should be collected, and how it can be used to assess the potential for transmission from treated to untreated individuals (docket number FDA-2014-D-0852). Finally, wild-type bacterial strains are difficult to obtain intellectual property protection for, making them unappealing for pharmaceutical companies to develop as they rely on patent protection to support their investment in medical research. For this reason, strains that are genetically modified for virulence attenuation and/or overexpression of therapeutic genes could be more attractive candidates for commercial investment. Successful resolution of some of the above issues would be a valuable early step toward the prospect of tumor-targeting bacteria entering clinical evaluation.

## 7. Concluding remarks

Despite immense progress, cancer often remains difficult to treat, suggesting that there is an urgent and unmet clinical need for novel and innovative therapies that will be efficacious where conventional therapies are not. The use of bacteria as immune stimulants, particularly in combination with established cancer immunotherapy strategies, has the potential to be part of a promising new frontier of immuno-oncology. Significant challenges remain in translating promising preclinical results into human clinical trials, but if successful, has the potential to change the lives of many cancer patients for whom immunotherapy alone is not effective.
